# Which Sámi? Sámi inclusion criteria in population-based studies of Sámi health and living conditions in Norway – an exploratory study exemplified with data from the SAMINOR study

**DOI:** 10.3402/ijch.v72i0.21813

**Published:** 2013-11-22

**Authors:** Torunn Pettersen, Magritt Brustad

**Affiliations:** 1 Centre for Sámi Health Research, Department of Community Medicine, University of Tromsø, Norway; 2 Department of Social Sciences, Sámi University College, Guovdageaidnu/Kautokeino, Norway

**Keywords:** indigenous, Sámi, Norway, ethnicity data, population-based study, inclusion criteria, health, living conditions

## Abstract

**Background:**

In a situation where national censuses do not record information on ethnicity, studies of the indigenous Sámi people's health and living conditions tend to use varying Sámi inclusion criteria and categorizations. Consequently, the basis on which Sámi study participants are included and categorized when Sámi health and living conditions are explored and compared differs. This may influence the results and conclusions drawn.

**Objective:**

To explore some numerical consequences of applying principles derived from Norway's Sámi Act as a foundation for formalized inclusion criteria in population-based Sámi studies in Norway.

**Design:**

We established 1 geographically based (G1) and 3 individual-based Sámi example populations (I1–I3) by applying diverse Sámi inclusion criteria to data from 17 rural municipalities in Norway north of the Arctic Circle. The data were collected for a population-based study of health and living conditions in 2003–2004 (the SAMINOR study). Our sample consisted of 14,797 participants aged 36–79 years.

**Results:**

The size of the individual-based populations varied significantly. I1 (linguistic connection Sámi) made up 35.5% of the sample, I2 (self-identified Sámi) made up 21.0% and I3 (active language Sámi) 17.7%. They were also noticeably unevenly distributed between the 5 Sámi regions defined for this study. The differences for the other characteristics studied were more ambiguous. For the population G1 (residents in the Sámi language area) the only significant difference found between the Sámi and the corresponding non-Sámi population was for household income (OR=0.69, 95% CI: 0.63–0.74). For the populations I1–I3 there were significant differences on all measures except for I2 and education (OR=1.09, 95% CI: 0.99–1.21).

**Conclusions:**

The choice of Sámi inclusion criterion had a clear impact on the size and geographical distribution of the defined populations but lesser influence on the selected characteristics for the Sámi populations relative to the respective non-Sámi ones.

Most of the indigenous Sámi people's traditional settlement area, Sápmi, corresponds to the central and northern parts of Norway, Sweden and Finland; a small portion covers the Kola Peninsula in north-western Russia. Studies of Sámi health and living conditions tend to use a variety of Sámi inclusion criteria and Sámi-internal categorizations (1–7). This may lead to uncertainty when the Sámi people's health and living conditions are studied and compared externally, internally, and over time.

Conceptually, indigenous peoples are also ethnic groups (8). Ethnicity is a complex phenomenon and it is not clear how and to what degree ethnicity is related to health (9,10). There is also uncertainty as to the appropriate, available and ethically acceptable procedures for obtaining the data needed to study health and ethnicity in interplay (11–16). Nevertheless, any study that relates to ethnically defined subpopulations must identify the groups in question. Thus, understandings and operationalizations of ethnicity as well as availability of relevant ethnicity data are crucial when designing population-based studies of indigenous people's health and living conditions.

A recent global survey found that 63% of all countries included some kind of “ethnic information” in their national censuses (17). This practice is thus widespread but not obvious. In the countries dividing Sápmi, there are various practices in time and space. For example, until 1930 many Norwegian and Swedish censuses did, in various ways and with various designations, identify Sámi citizens; after 1945 this has only happened as a few geographically limited exceptions (18–20). Despite some inter-country variations, the present situation is a fundamental lack of systematic up-to-date Sámi demographic data. A part of this situation is, however, that the Sápmi area historically has been the home of several ethnic groups. Although there have been hierarchies in terms of power and status, including periods of forced assimilation of the Sámi, the various groups have also interacted (21–23). The foundation for Sámi ethnicity and the basis for individuals’ self-identification as Sámi is thus ambiguous. Hence, even if information on Sámi affiliation had been systematically collected in official contexts, it is not given which persons could and would have been recorded as Sámi.

An absence of ethnicity data in national censuses implies that official ethnic categories are also absent. However, in Norway a special Sámi Act passed in 1987 has stated some criteria for the right to participate in elections to a national Sámi representative body – *the Sámediggi* in Northern Sámi. The act also states the right to use Sámi language in certain contexts. In this study, we wanted to explore the possibility of utilizing principles derived from this act as a foundation for formalized inclusion criteria in Sámi population-based studies in Norway. The aim was to define Sámi example populations based on these principles and to present some numerical consequences of this. We also aimed to determine whether different definitions would provide different effects when the outcomes on 3 measures related to health and living conditions were compared for each corresponding Sámi and non-Sámi population.

## Materials and methods

### Data and study population

**Fig. 1:**
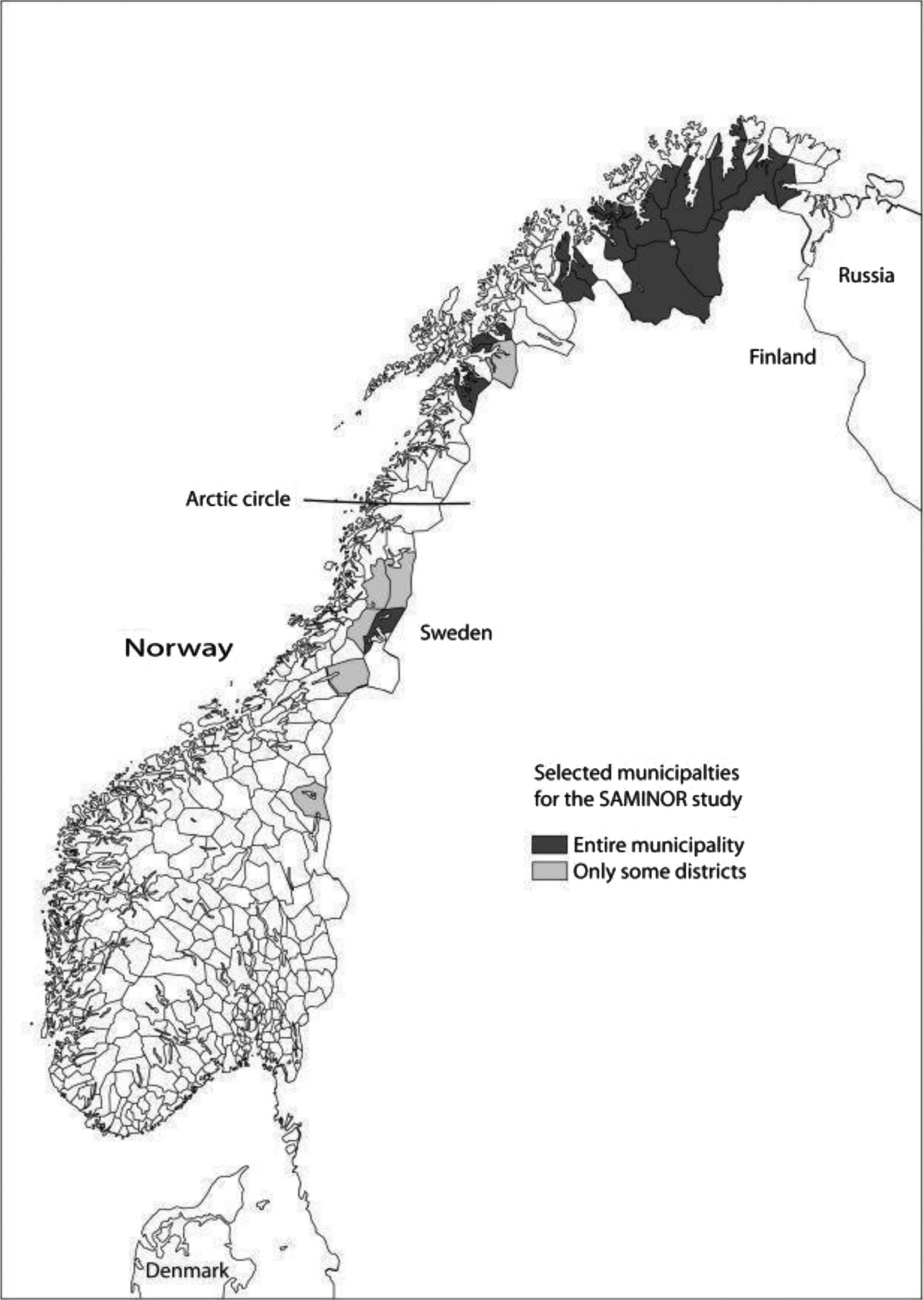
Municipalities included in the SAMINOR study.

We used data collected in 2003–2004 for the SAMINOR study, a population-based cross-sectional study of health and living conditions in selected rural and semi-rural areas in the Norwegian part of Sápmi, where available knowledge indicated the presence of some Sámi population (24). The SAMINOR study was initiated by the Centre for Sámi Health Research at the University of Tromsø and was conducted in collaboration with the Norwegian Institute of Public Health. The study included 24 municipalities, 18 north of and 6 south of the Arctic Circle. In 7 municipalities, the study was however limited to certain villages (Fig. 1).

In total 27,987 persons living in the selected areas, aged 36–79 years, were invited to participate. Among these, 60.6% returned at least one of the study's three questionnaires. Our study included 14,797 participants aged 36–79 years who (a) were resident north of the Arctic Circle in 1 of the 17 entirely involved municipalities, and (b) had returned the questionnaire including the questions about ethnicity. As of 1 January 2003, the total population in our study area amounted to 1.1% of Norway's total population.

### Sámi example populations

We defined a set of Sámi populations based on principles derived from the Sámi Act) (25). This act states that the Sámi in Norway shall have a nationwide Sámi representative body, elected by and among those Sámi who have joined a separate electoral roll established for this purpose. The right to enrol requires that a person declares to fulfil *both* a subjective criterion of self-identification as Sámi *and* an objective criterion saying that the person or at least 1 parent, grandparent or great-grandparent has or has had Sámi as a language at home, or that the person is the child of an enrolled person (Section 2-3). Since 1990 the Sámi Act has given individuals the right to use Sámi language in certain contexts, primarily in municipalities designated as the *Sámi Language Administrative District* (Section 3-1). This area – hereafter the *Language Area* – originally comprised 6 municipalities, but has, since 2006, gradually been extended to 10, 8 of which are in our study area.

There appear to be 2 premises to be derived from the Sámi Act: that Sámi identification shall be self-ascribed, and that the Sámi language has a particular status as a basis for Sámi rights. The latter relates to geography (*cf*. the Language Area) and to the individual – to persons who speak Sámi (*cf*. the right to use the Sámi language) and to persons whom this was the case for at least 1 person in the 3 previous generations (*cf*. the objective criterion for enrolment in the Sámediggi electoral roll). We synthesized the 2 premises into 3 principles which we considered to be a salient basis for defining Sámi populations, namely (a) geographical location, (b) linguistic connection and (c) ethnic self-identification.

The SAMINOR study was conducted in municipalities with a minimum proportion of Sámi residents. The entire study sample might thus be regarded as a kind of geographically based Sámi population (G0). However, with reference to the Sámi Act, we defined 4 more or less overlapping Sámi populations: 1 geographically based (G1) and 3 individual-based (I1–I3). The geographical population G1 consists of all participants resident in the municipalities included in the Language Area in 2013. In our study area these are Nesseby, Tana, Porsanger, Karasjok, Kautokeino, Kåfjord, Lavangen and Tysfjord – listed from northeast towards south, Norwegian names only. The participants’ geographical affiliation was stated in address data obtained from the Norwegian National Population Register.

Two individual-based Sámi populations, I1 and I2, were defined with reference to the objective and the subjective criterion for enrolment in the Sámediggi electoral roll. Population I1 consists of individuals who reported any kind of Sámi linguistic connection in 3 generations (the criterion in the Sámi Act also includes great-grandparents’ language, but our data only cover 3 generations). Population I2 consists of those who reported self-identification as Sámi. A third individual-based population I3 is composed of those who reported Sámi as an active language. Assignment to I1–I3 was based on self-reported replies to the 2 SAMINOR questions (a) What language do or did you, your parents, and your grandparents use at home? and (b) What do you consider yourself? For all questions, one or more boxes could be ticked for the options “Norwegian,” “Sámi,” “Kven” and “Other, please describe” (in our study area “Kven” represents descendants of Finnish pre-1945 immigrants, now formally recognized as a national minority in Norway). The responses about language were to be specified for each parent and grandparent. We categorized the person/language as Sámi when the Sámi option was ticked, either alone or combined with one or more other options.

We also defined 2 non-Sámi populations (N0 and N1) consisting of participants who had *not* ticked the Sámi option for *any* of these questions. Population N0 covers those in the entire study area whereas N1 are restricted to those in the Language Area. The non-Sámi thus does not constitute an ethnic group but represent statistical populations compiled for analytical purposes.

### Demographic variables

**Fig. 2:**
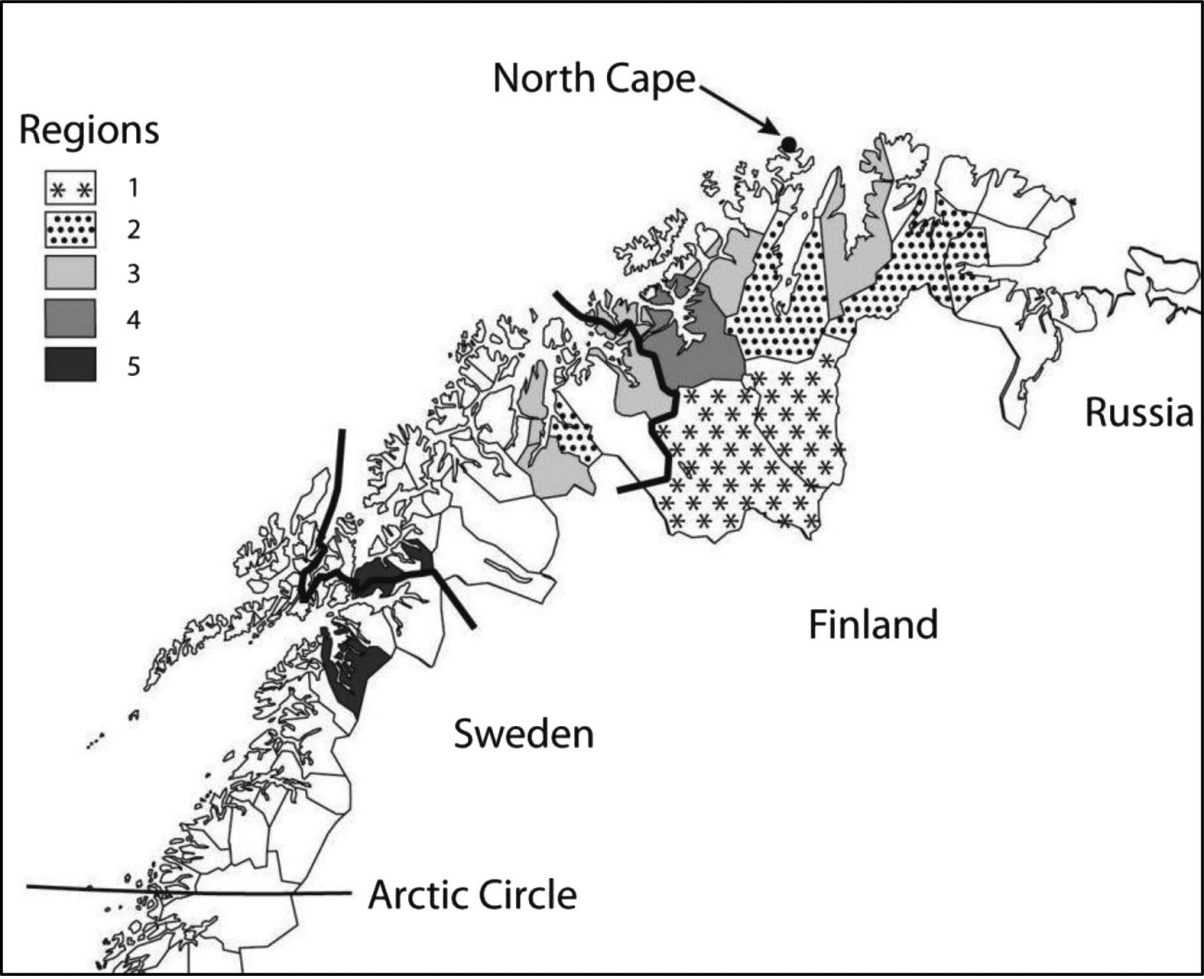
Sámi regions defined for this study*. *Detailed Region labels: 1, Inner Language Area (2 municipalities); 2, Outer Language Area (4 municipalities); 3, Areas of Northern Troms/Finnmark (6 municipalities); 4, Alta municipality; 5, Areas of Nordland/Southern Troms (4 municipalities). The 6 municipalities in Region 1 and 2 together with 2 of those in Region 5 (the southernmost and the northernmost) make up those 8 municipalities in the Sámi Language Administrative District in 2013 that are included in our study area.

We used data on gender and age provided by the Norwegian Central Population Register. We divided the variable “Age” into the categories “36–48 years,” “49–61 years” and “62–79 years.” To obtain a more detailed picture of each population's geographical distribution, we constructed the variable “Region” where the 17 municipalities were grouped into 5 regions, based primarily on Sámi cultural distinctions but also on location and population size (Fig. 2).

Six municipalities that make up the original Language Area constitute Region 1 'Inner Language Area’ (Kautokeino and Karasjok) and Region 2 'Outer Language Area’ (Kåfjord, Porsanger, Tana and Nesseby). This distinction was made because the Sámi language during recent decades has had a stronger position in the 2 municipalities of Region 1. Six municipalities with traditional coastal Sámi settlement constitute Region 3 'Areas of Northern Troms/Finnmark’ (Storfjord, Lyngen, Kvænangen, Loppa, Kvalsund and Lebesby). Region 4 ‘Alta’ covers only 1 municipality, Alta, which is also on the coast but because of a large and expanding population declared town status in 2000. Region 5 'Areas of Nordland/Southern Troms’ consists of 4 municipalities with Lule Sámi (Tysfjord) and Marka (i.e. outlying fields in the inland area) Sámi settlements (Evenes, Skånland and Lavangen). Tysfjord and Lavangen were included in the Language Area in 2006 and 2009, respectively.

### Measures related to health and living conditions

We used SAMINOR questions to construct 3 measures widely used in studies of health and living conditions, namely education, household income and self-reported health. “How many years of education have you completed?” was divided into the categories “less than 12 years” and “more than 12 years,” corresponding to maximum completed high school and minimum commenced higher education, respectively. “How big is the family/household gross income per year?” had originally 6 response categories, but we constructed a dichotomous variable “Household income” with a cut-off point at 300,000 NOK. “Self-rated health” was dichotomized into “good” and “not good,” based on “How is your health now?”, which originally had 4 options.

### Statistical analyses

Statistical analyses were performed in STATA, Version 12. Frequency tables were used for descriptive analyses. Logistic regression was used to compare (dichotomized) outcomes for the 3 measures related to health and living conditions for each Sámi and the corresponding non-Sámi population defined by the various Sámi inclusion criteria.

### Ethics

The SAMINOR study was approved by the Regional Ethics Committee for Medical Research in Northern Norway. A Sámi consultant participated in the review of the application. Permission for retention of personal data was provided by the Norwegian Data Inspectorate. Beyond this, in contrast to many other indigenous peoples, the Sámi have not adopted Sámi-specific guidelines or procedures for research involving Sámi participants (26).

## Results

**Fig. 3:**
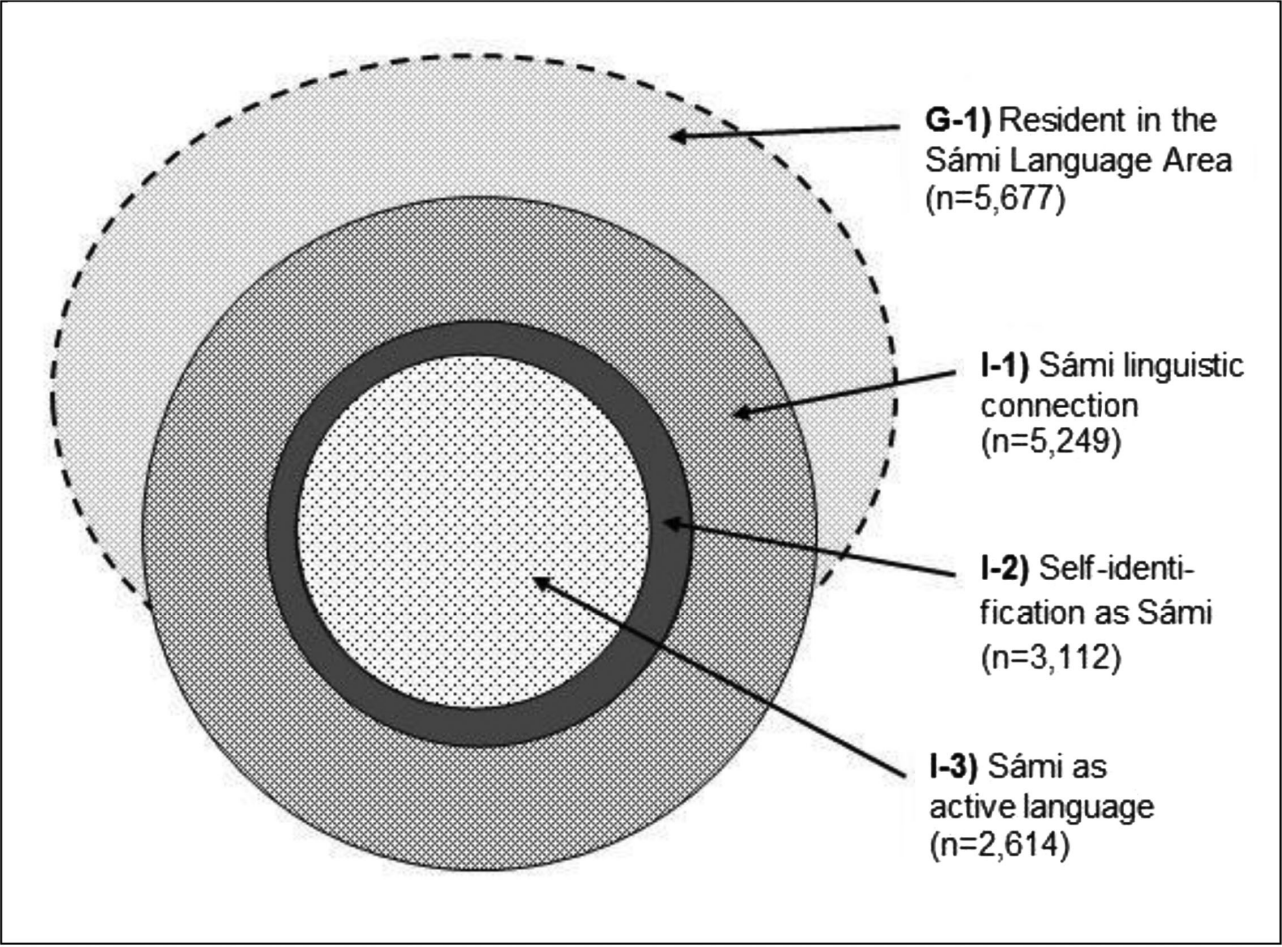
Schematic illustration of 4 Sámi populations aged 36–79 years as of 2003–2004 in 17 municipalities in Norway north of the Arctic Circle – defined according to different but partially overlapping Sámi inclusion criteria*. *The size and relative position of each circle is indicative. Assignment to I1–I3 is based on self-reported data.

Table I presents the absolute and relative size of the defined populations. Those with Sámi linguistic connection were twice as many as those reporting Sámi as the active language. The number of self-identified Sámi was somewhat higher than that of active-language users. Additional calculations show that among linguistic connection, 59.3% reported Sámi self-identification and 49.8% reported Sámi as the active language. Among the self-identified Sámi 83.9% reported being a Sámi speaker. Those residents inside the Language Area and with negative reporting on all Sámi inclusion criteria made up 39.1% of all respondents in this area. Figure 3 gives a schematic illustration of the 4 defined Sámi populations.

**Table I:** Five Sámi and 2 non-Sámi populations aged 36–79 years as of 2003–2004 in 17 municipalities in Norway north of the Arctic Circle – defined according to different but partially overlapping Sámi inclusion/exclusion criteria*

	n	%
Geographically based Sámi populations		
G0) Resident in the study area (17 municipalities)	14,797	100.0
G1) Resident in the Language Area (8 municipalities)	5,677	38.4
Individual-based Sámi populations		
I1) Sámi linguistic connection objective criterion#	5,249	35.5
I2) Self-identification as Sámi=subjective criterion	3,112	21.0
I3) Sámi as active language	2,614	17.7
Non-Sámi populations – geographically and individual-based		
N0) Resident in the study area – no reported Sámi inclusion criteria, *cf*. G0	9,409	63.9
N1) Resident in the Language Area – no reported Sámi inclusion criteria, *cf*. G1	2,220	39.1

* Assignment to I1–I3 and N0–N1 is based on self-reported data. Due to missing values 60 participants were included neither in I1–I3 nor in N1–N2. Percentages for these populations take into account the missing values.

# The criterion for enrolment in the Sámediggi electoral roll applies to 4 generations, our data cover 3.

Characteristics of the 5 Sámi populations are shown in Table II. The gender and age distributions were fairly uniform, but those with Sámi as the active language had a slightly higher proportion of the oldest category at the expense of the youngest.

**Table II:** Distribution of characteristics of 5 Sámi populations aged 36–79 years per 2003–2004 in 17 municipalities in Norway north of the Arctic Circle – defined according to different but partially overlapping Sámi inclusion criteria

	Based on geography – residence in a Sámi settlement area				Based on the criteria for enrolment in the Sámediggi electoral roll				Based on the use of Sámi language	
			
	G0) Resident in the study area – 17 municipalities (n=14,797)		G1) Resident in the Language Area – 8 municipalities (n=5677)		I1) Sámi linguistic connection objective criterion# (n=5249*)		I2) Self-identification as Sámi=subjective criterion (n=3112*)		I3) Sámi as active language (n=2614*)	
					
	n	%	n	%	n	%	n	%	n	%
Gender										
Men	7,162	48.4	2,773	48.9	2,641	50.3	1,541	49.5	1,309	50.1
Women	7,635	51.6	2,904	51.2	2,608	49.7	1,571	50.5	1,305	49.9
Age										
36–48 years	4,998	33.8	1,916	33.8	1,757	33.5	1,089	35.0	797	30.5
49–61 years	5,749	38.9	2,205	38.8	2,035	38.8	1,196	38.4	998	38.2
62–79 years	4,050	27.4	1,556	27.4	1,457	27.8	827	26.6	819	31.3
Region										
1 Inner Language Area (2 municipalities)	1,507	10.2	1,507	26.6	1,309	24.9	1,245	40.0	1,204	46.1
2 Outer Language Area (4 municipalities)	3,370	22.8	3,370	59.4	1,928	36.7	1,135	36.5	967	37.0
3 Areas of Northern Troms/Finnmark (6 municipalities)	3,240	21.9	–	–	901	17.2	232	7.5	123	4.7
4 Alta muni.	4,626	31.3	–	–	770	14.7	255	8.2	151	5.8
5 Areas of Nordland/Southern Troms (4 municipalities)	2,054	13.9	800	14.1	341	6.5	245	7.9	169	6.5
Self-reported length of education**										
≤12 years	8,854	67.4	3,119	68.0	3,066	70.4	1,607	65.4	1,418	71.2
>12 years	4,274	32.6	1,467	32.0	1,292	29.7	851	34.6	573	28.8
Self-reported household income**										
≤Kr. 300,000	5,151	38.6	2,209	43.4	2,156	45.7	1,291	46.0	1,156	50.0
>Kr. 300,000	8,181	61.4	2,886	56.6	2,559	54.3	1,514	54.0	1,157	50.0
Self-rated health**										
Not good	4,537	32.8	1,597	33.1	1,637	35.5	889	34.3	761	35.9
Good	9,308	67.2	3,232	66.9	2,973	64.5	1,703	65.7	1,361	64.1

* Assignment is based on self-reported data.

# The criterion for enrolment in the Sámediggi electoral roll applies to 4 generations, our data cover 3.

** Varying n because of missing values.

The geographical variations were far more striking. The linguistic connection-based population was the only one with the largest share settled in Region 2 Outer language area. This population was also somewhat lesser unevenly distributed than the 2 other individual-based populations, whose patterns of regional distribution were fairly similar. In the population defined according to Sámi as the active language, 83.1% was resident in Region 1 or Region 2, the inner and the outer Language area.

With respect to household income, the proportion having the most favourable outcome sank gradually from 61.4 to 50.0% along the “population axis” G0–I3. Similar trends were revealed for education and self-rated health but with smaller relative differences. However, for education the pattern was broken by the self-identified Sámi having the largest proportion with the better outcome.

Characteristics of the 2 non-Sámi populations (Table III) had similar patterns to those of the 2 geographically based populations, respectively (*cf*. Table II). The geographical variations were, however, even greater and for household income the non-Sámi populations had a slightly larger proportion of those with the better outcome.

**Table III:** Distribution of characteristics of 2 non-Sámi populations aged 36–79 years as of 2003/2004 in 17 municipalities in Norway north of the Arctic Circle – defined according to not having reported self-identification as Sámi or Sámi linguistic connection within 3 generations*

	N0) Resident in the study area – 17 municipalities (n=9,409)		N1) Resident in the language area – 8 municipalities (n=2,220)	
		
	n	%	n	%
Gender				
Men	4,435	47.1	1,040	46.9
Women	4,974	52.9	1,180	53.2
Age				
36–48 years	3,196	34.0	708	31.9
49–61 years	3,656	38.9	871	39.3
62–79 years	2,557	27.1	641	28.9
Region				
1 Inner Language Area (2 municipalities)	175	1.9	175	7.9
2 Outer Language Area (4 municipalities)	1,394	14.8	1,394	62.8
3 Areas of Northern Troms/Finnmark (6 municipalities)	2,314	24.6	–	–
4 Alta municipalities	3,832	40.7	–	–
5 Areas of Nordland/Southern Troms (4 municipalities)	1,694	18.0	651	29.3
Self-reported length of education**				
≤12 years	5,731	66.1	1,260	65.9
>12 years	2,943	33.9	651	34.1
Self-reported household income**				
≤Kr. 300,000	2,952	34.7	749	37.7
>Kr. 300,000	5,568	65.4	1,236	62.3
Self-rated health**				
Not good	2,872	31.5	660	32.9
Good	6,248	68.5	1,345	67.1

* Due to missing values, 60 participants were included neither in the populations I1–I3 nor in N1–N2.

** Varying n because of missing values.

Table IV presents odds ratios (OR) and 95% confidence interval (CI) for education, household income and self-rated health for each of the populations G1 and I1–I3. The respective OR and CI are calculated by using as reference group each population's respective non-Sámi proportion of the study sample. All measures are adjusted for gender and age. For household income, the members of all the defined Sámi populations had significant – and markedly – lower odds for better outcome than the respective non-Sámi populations. Also self-rated health displayed a less favourable result for the 4 Sámi populations. The finding was however not significant for the population comprising all those in the Language Area (OR=0.98, CI: 0.91–1.06). The most diverse findings were those concerning the odds of having higher education. Whereas definitions based on Sámi linguistic connection and Sámi as the active language resulted in lower odds for Sámi versus non-Sámi, there was no significant difference between Sámi and non-Sámi population when using the definition based on geography, that is, all in the Language Area (OR 0.96 and CI 0.89–1.05) and the definition based on self-identification as Sámi (OR 1.09 and CI 0.99–1.21).

**Table IV:** Adjusted odds ratio (OR) and 95% confidence interval (CI) for education, household income and self-rated health when 4 partially overlapping Sámi populations aged 36–79 years as of 2003–2004 in 17 municipalities in Norway north of the Arctic Circle are related to each population's respective non-Sámi proportion of the study sample*

	OR	95% CI	p
Self-reported length of education: >12 vs. ≤12 years			
G1) Resident in the Language Area – 8 municipalities (n=5,677)	0.96	0.89–1.05	0.386
I1) Sámi linguistic connection≈objective criterion# (n=5,249*)	0.81	0.75–0.88	**
I2) Self-identification as Sámi=subjective criterion (n=3,112*)	1.09	0.99–1.21	0.070
I3) Sámi as active language (n=2,614*)	0.87	0.78–0.97	***
Self-reported household income: >300,000 vs. ≤300,000 NOK			
G1) Resident in the Language Area – 8 municipalities (n=5,677)	0.69	0.63–0.74	**
I1) Sámi linguistic connection≈objective criterion#3 (n=5,249*)	0.57	0.53–0.62	**
I2) Self-identification as Sámi=subjective criterion (n=3,112*)	0.60	0.55–0.67	**
I3) Sámi as active language (n=2,614*)	0.55	0.50–0.61	**
Self-rated health: good vs. not good			
G1) Resident in the Language Area – 8 municipalities (n=5,677)	0.98	0.91–1.06	0.641
I1) Sámi linguistic connection≈objective criterion# (n=5,249*)	0.83	0.77–0.89	**
I2) Self-identification as Sámi=subjective criterion (n=3,112*)	0.89	0.81–0.98	***
I3) Sámi as active language (n=2,614*)	0.88	0.80–0.98	***

* Assignment to I1–I3 is based on self-reported data. All OR adjusted for sex and age and take into account that n varies because of missing values.

# The criterion for enrolment in the Sámediggi electoral roll applies to 4 generations, our data cover 3.

** p<0.0001

*** p<0.05.

When testing the heterogeneity of the *individual*-based Sámi inclusion criteria separately (I1–I3, *cf*. Table IV), we found statistically significant difference only for education between self-identified Sámi and the 2 other populations respectively, whereas heterogeneity testing including the *geographically* defined population (G1) revealed a higher degree of heterogeneity between the different populations (data not shown).

## Discussion

We found that the size and geographical distribution of a Sámi study population were noticeably affected by Sámi inclusion criterion. The differences for the other characteristics studied were more varied. A proportion of about 40% self-reported non-Sámi in the Language Area is also noteworthy. The findings must be viewed in light of Norway's assimilation policy, the so-called Norwegianization. From about 1850 to about 1950–1980, depending on the definition, this policy entailed a systematic governmental effort to make Sámi give up their language, change the basic values of their culture and replace their Sámi identity (23). The policy was by and large successful; many who could have been Sámi speakers and/or self-identified as Sámi did gradually loose or drop these identification markers, particularly in coastal areas (27–29).

Although the modern Sámi movement in Norway during the 1970s led to a gradual change towards the objective that no one (any longer) should deny, conceal or give up Sámi identity and language (30), the historical legacy of the Norwegianization is still manifest. This is demonstrated by the fact that self-identified Sámi in this study was equivalent to about 60% of linguistic connection Sámi. But while not self-identifying as Sámi despite having a Sámi linguistic connection might indicate that Sáminess (still) carries a social stigma, in some communities more than others, it is also widely agreed upon that ethnicity and ethnic affiliation are complex phenomenons which over time have been understood and dealt with differently by both scholars and laymen. Thus, to not self-identify as Sámi might for some individuals be based on rational assessment; they could have been Sámi, but do not consider themselves as such because their way of living does not coincide with their understanding of “what it means to be Sámi” (31–37). Their “objective” Sámi connection does anyway persist.

Those who reported Sámi as active language made up the smallest population. However, this group might be of particular interest – with respect to linguistic accommodation of health services for the Sámi-speaking population in a specific area and also, to studies of this topic. Besides, to define the speakers of Sámi language as a distinct population takes into account the possibility of being a Sámi speaker without having Sámi ancestry.

Whereas the use of individual-based Sámi inclusion criteria requires access to self-reported data, the use of geographical criteria is more straightforward. Pragmatically this might be a tempting alternative since much data can be obtained from regular municipality statistics. However, analyses and interpretations must take into account that a geographically defined Sámi population inevitably will have a non-Sámi share; in our study 39.1% in the Language Area. Also, the use of geographical criteria excludes the experiences of Sámi who live in communities with less Sámi-ethnic density, in itself a matter of interest.

Studies combining ethnicity and health-related issues do often have *between-group* equality and equity as focal aspects. In our study the geographical definition of a Sámi population implied a significant difference between Sámi and non-Sámi populations for household income only. The individual-based definitions resulted in significant differences on all measures except on education for self-identified Sámi. The significant differences were all to the disadvantage of the Sámi, though only to a minor extent with respect to education and self-rated health. However, while between-group differences clearly are of interest for Sámi and other indigenous peoples, it is also essential to have knowledge of each indigenous people's health and living conditions *per se*. Such knowledge is necessary as a means for the implementation of the principles in the UN Declaration on the rights of indigenous peoples (38). This includes knowledge about within-group differences *among Sámi*; whether and how Sámi health varies with affiliation to various Sámi communities and also, with the belonging to *other* social groups than the ethnic one (39).

Scholars have pointed out that studies of health and ethnicity tend to underestimate or under-communicate that ethnicity is context-dependent and invokes explicit and articulated operationalization in each case (40–42). In fact, failing to provide the context and definitions of study populations is claimed to be a common mistake in studies of health patterns at the population level (43). This is problematic because it can affect the calculations of interest, the understanding of causal relationships, and also the potential for generalization (44). Thus, when health studies include ethnic populations it is particularly important – and maybe also particularly challenging – to articulate the demographic frameworks and to specify the analytic categories. This is perhaps even more essential in studies involving indigenous peoples (45–47). Our study contributes to on-going efforts to deal with such challenges in a Sámi context.

We would argue that a key challenge for population-based Sámi studies is to manage the relationship between the inclusion criteria based on Sámi linguistic connection and self-identification as Sámi. One aspect is that these 2 populations differ in several respects, especially geographically. But it is also important that it seems to have become (ethically) preferable to base ethnicity data on self-identification (48). For instance, the United Nations explicitly recommends self-identification when ethnicity is recorded in a national census, including also the possibility of multi-ethnic identification (49). However, because some persons who do not (any longer) identify with a particular ethnic group still might be influenced by having a (former) connection to this group, it may be appropriate to combine “subjective” data based on self-identification with data based on one or more “objective” manifestations of ethnic affiliation, even though reporting of the latter may also be unstable. Dilemmas arising from incorporating and presenting individuals as something “other” than what they consider themselves to be should however be explicitly addressed.

## Limitations

The Sámi data situation implies that it is not possible to assess whether there are ethnic biases in the sample. The self-reported Sámi ethnicity data must be related to that all SAMINOR participants were born before 1969 and thus might have life experiences affected more by the Norwegianization of the past than by the Sámi revitalization of more recent decades. We did not take into account that some respondents had ticked for more than one option when answering the ethnicity questions. Neither did we consider possible inconsistencies in the reporting.

## Conclusion

Every population-based Sámi study, whether having a Sámi/non-Sámi “dichotomy perspective” or a Sámi-internal “gradient perspective”, has to decide on how to define participants as Sámi participants. Our pioneering exploration of the utilization of formalized Sámi inclusion criteria demonstrated that the choice of criterion affected the size and geographical distribution of the various Sámi populations. The impact of the criterion on the selected characteristics for each of the Sámi populations relative to the respective non-Sámi ones was small, though not absent. Generally speaking, however, any study of Sámi health and living conditions would benefit from a transparent assessment of utilizing formalized Sámi inclusion criteria.
